# Association between Physical Fitness and Perceived Well-Being in Functionally Independent Community Dwelling Elderly of North-Eastern India

**DOI:** 10.15388/Amed.2023.30.1.1

**Published:** 2023-01-24

**Authors:** Prasanta Kumar Bhattacharya, Kuldeep Deka, Bhupen Barman, Md Jamil

**Affiliations:** Department of General Medicine, North Eastern Indira Gandhi Regional Institute of Health and Medical Sciences (NEIGRIHMS), Shillong-793018, India; Department of Physiotherapy, Down Town Hospital, Guwahati-781005, India; Department of General Medicine, North Eastern Indira Gandhi Regional Institute of Health and Medical Sciences (NEIGRIHMS), Shillong-793018, India; Department of General Medicine, North Eastern Indira Gandhi Regional Institute of Health and Medical Sciences (NEIGRIHMS), Shillong-793018, India

**Keywords:** Physical fitness, Perceived well-being, Community dwelling elderly

## Abstract

**Background:**

Regular physical activity helps in independent living, prevention of chronic health problems and quality of life in the elderly. The aim of the study is to determinewhether physical fitness is associated with multiple dimensions of well-being in the community dwelling elderly.

**Materials and Methods:**

A community-based cross-sectional study was undertaken to assess the physical fitness and perceived wellness in 400 elderly (≥65 years) subjects. The Senior Fitness Test (SFT) for assessing functional/physical fitness and Perceived Wellness Survey (PWS) were used to assess their well-being. Bivariate correlation analysis was used for individual testsand multiple linear regressions were used to analyze relationship of wellness composite score with physical fitness test.

**Results:**

284men and 116 women (mean ages 69.80±3.82 and67.25±2.57 years, respectively) were assessed for physical fitness tests and perceived wellness.‘Arm-curl’ and ‘chair-sit’ testsshowedlinear decrease in strength with increasing age. In ‘back-scratch’ and ‘chair-sit and reach’ tests lower limb flexibility was better than upper limb in all except the 65–69 year sub-group. Maximum time to perform the ‘8-feet up-and-go’ test increased progressively with age, whereas ‘2-minute step’ test showed a linear decrease in mean score with advancing age. Wellness composite score (14.54±2.31) inmaleswas maximum in the 75–79 year age-group, while in females it (15.26±2.29) was maximum in the 70–74 year age-group. Correlation analysis of physical fitness test with perception of wellness (composite score) showed significant association of ‘arm-curl’ test (p=0.012), ‘back-scratch’ test (p=0.0002), ‘8-feet up-and-go’ test (p=0.005), ‘2-minute step’ test (p=0.005) with the composite wellness score in the male participants, whereas in the females such significance was observed only in the ‘2 minute step’ test (p=0.007) with the wellness score.

**Conclusion:**

Screening of physical fitness and wellness are important measures in assessing wellness of community dwelling elderly, and in predicting theiroverall state of well-being, including age-specific comparison of fitness performance and wellness score.

## Santrauka.

**Įvadas:**

Reguliari fizinė veikla padeda pagyvenusiems žmonėms gyventi savarankiškai, yra prevencinė lėtinių sveikatos problemų priemonė ir užtikrina jų gyvenimo kokybę. Šio tyrimo tikslas – nustatyti, ar bendruomenėje gyvenančių pagyvenusių žmonių fizinis tvirtumas gali būti siejamas su daugeliu gerovės dimensijų.

**Medžiaga ir metodai:**

Atliktu bendruomenės narių skerspjūvio tyrimu siekta įvertinti 400 pagyvenusių tiriamųjų (≥65 metai) fizinį tvirtumą ir subjektyviai suvokiamą savijautą. Senjorų fizinio tvirtumo testu (SFT) įvertinta tiriamųjų funkcinė ir fizinė būklė, o subjektyviai suvokiamos gerovės apklausa (PWS) buvo pasitelkta įvertinti jų gerovę. Atlikus bivariacinės koreliacijos tyrimą buvo įvertinti konkretūs tyrimai, o daugialypė linijinė regresija naudota sudėtinio gerovės balo sąsajai su fizinio tvirtumo testo rezultatais ištirti.

**Rezultatai:**

Tyrime dalyvavo 284 vyrai ir 116 moterų (vidutinis vyrų amžius 69,80±3,82 metų, moterų – 67,25±2,57 metų). Atliktas tiriamųjų fizinės gerovės testas ir išsiaiškinta jų subjektyviai suvokiama savijauta. Rankos lenkimo testas (kai sėdint reikia kuo daugiau kartų pakelti svarmenį lenkiant ranką) ir sėdėjimo kėdėje testas atskleidė linijinį fizinio stiprumo mažėjimą didėjant amžiui. Nugaros pasikasymo bei sėdėjimo kėdėje ir galūnių pasiekimo testai leido nustatyti, kad visų tyrimo dalyvių, išskyrus 65–69 metų grupę, apatinių galūnių lankstumas yra geresnis nei viršutinių galūnių. Maksimalus laikas atliekant testą „2 m 40 cm: atsistoju ir nueinu“ progresyviai didėjo su amžiumi, o „žingsnių per dvi minutes“ testas atskleidė linijinį vidurkio mažėjimą augant amžiaus vertei. Sudėtinis fizinio tvirtumo rezultatas (14,54±2,31) vyrų buvo geriausias 75–79 metų amžiaus grupės, o moterų šis rezultatas geriausias (15,26±2,29) 70–74 metų amžiaus grupės. Fizinio tvirtumo testo ir subjektyviai suvokiamos savijautos testo koreliacinė analizė (sudėtinis balas) parodė, kad vyrų sudėtinė gerovės vertė reikšmingai susijusi su rankos lenkimo testu (p = 0,012), nugaros kasymosi testu (p = 0,0002), „2 m 40 cm: atsistoju ir nueinu“ testu (p = 0,005) bei žingsnių per dvi minutes testu (p = 0,005), o moterų reikšminga sąsaja nustatyta tik subjektyviai suvokiamos gerovės ir žingsnių per dvi minutes testo (p = 0,007).

**Išvada:**

Fizinio tvirtumo ir gerovės vertinimo testai yra svarbūs vertinant pagyvenusių žmonių bendruomenę ir nustatant jų bendrąjį gerovės lygmenį, įskaitant tarpusavio palyginimą siejant fizinės gerovės lygmenį tam tikru amžiaus tarpsniu ir subjektyviai suvokiamos gerovės rezultatą.

## Introduction

With the improvement in healthcare, decline in fertility and increasing life expectancy, the number of elderly people worldwide is projected to increase by over 20% of the total population by 2050 [1,2]. This trend would continue even in the developing countries; for example, in India, the elderly population which accounted for 5.6% of the total population in 1961 is expected to increase over 12.4% by the year 2026 [1].

A major problem associated with advanced age is a marked decline in functional capacity and an associated loss of independence [3]. However, successful ageing is multi-dimensional, encompassing the avoidance of disease and disability, the maintenance of high physical and cognitive function and sustained engagement in social and productive activities [4]. It has been demonstrated that in the elderly, the overall perception of well-being, self-esteem, emotional well-being, self-concept, happiness and life satisfaction are being affected by physical activity [5,6]. Physical exercise is a subcategory of physical activity that is planned, structured and repetitive with the objective of improvement or maintenance of physical fitness [7]. Participation in physical activity and exercise can contribute to maintaining quality of life, health, physical function and reducing falls among the elderly in general and with morbidities [8-10]. Several studies have investigated the role of objective dimensions such as physical health[11], social support from family and friends [12], free time investment [13,14] and physical activity [15] as predictors of successful ageing [16].While sufficient research has been done on the morbidity pattern and functioning in elderly is available, there is very limited data on the physical related fitness and multiple dimensions of wellness among the elderly population in India. Since there is an increase in the elderly population in India, research in this area is important. Therefore, the purpose of this research is to examine how physical related fitness is associated with multiple dimensions of well-being in a cohort of community dwelling elderly.

## Materials and Methods

A community based cross-sectional study was undertaken to assess the physical fitness and perceived wellness in 400 elderly subjects, aged ≥65 years during the period from January 2016 to August 2020. Assuming aprevalence rate of elderly of 50% at 95% confidence interval, with a precision rate of 5%, the sample size was found to be 400, using the formula 4pq/I^2^where p = prevalence, q = 1-p and I= standard error.

Participants were selected from the community in the municipality blocks in Guwahati, the largest city in the northeast region of India. Eighteen of the 60 municipal wards were selected by random sampling; households from each ward were selected by systematic sampling and one participant, either male or female, from each selected household was included based on the inclusion and exclusion criteriafor their physical fitness and perceived wellness.Based on their age, participants were divided into four subgroups: 65–69 years, 70–74 years, 75–79 years and ≥80 years.

*Inclusion criteria:* all functionally independent elder people over the age of 65 years, of either gender with no physical or cognitive limitation that would prohibit them to follow instructions or to participate safely, were included.

*Exclusion criteria:* Those with acute illnesses, unstable musculoskeletal injury, uncontrolled hypertension, uncontrolled or end stage systemic diseases and impaired vision hampering mobility or test performance were excluded from the study.

The study was approved by the Institutional Ethical Committee of Gauhati Medical College and written informed consent for screening with Physical Activity Readiness Questionnaire (PAR-Q) was obtained from all subjects before the enrolment in the study. Following which, socio-demographic characteristics and height (in centimeters), weight (in kilograms) measurement were taken to calculate the body mass index (BMI).

### Outcome variables

**Senior Fitness Test:** The main outcome was the Senior fitness test (SFT) which is convenient for safe assessment in providing information about physical fitness in elderly people [17,18]. The SFT was designed to assess underlying parameters associated with functional mobility such as muscle strength, cardio-respiratory endurance, flexibility, balance and agility/mobility [19]. Each item was developed and validated as a means of assessing the underlying physical attributes that support functional mobility. Test-retest reliability for SFT items ranged from .80 to .98. Validity was established through various types of content and criterion analyses, including comparing SFT scores with other “gold standard” measures, such as Treadmill VO2 Max testing and one repetition maximum strength testing [20].The SFT assessments were made in the order presented as shown in Table 1.Each test of the SFT [21] was first demonstrated to the participants and if necessary, cues or gestures were provided. Participants were instructed and encouraged to do the best they can on all the tests, but never to push themselves to a point of overexertion or beyond what they think is safe for them. All participants performed the six physical tests in their own residences.

**Table 1.** Methodology of Senior Fitness Test [17]

**Assessment category**	**Test item**	**Test description**
Upper body Strength	Arm curl test	Number of bicep curls that can be completed in 30 seconds holding a hand weight – 5 pounds for women; 8 pounds for men.
Lower body Strength	Chair stand test	Number of full stands from a seated position that can be completed in 30 seconds with arms folded across chest.
Upper body flexibility	Back scratch test	With one hand reaching over the shoulder and one up the middle of the back, the number of inches (centimetres) between the extended middle fingers (plus or minus).
Lower body flexibility	Chair sit-and-reach test	From a sitting position at the front of a chair, with leg extended and hands reaching toward toes, the number of inches (centimetres) (plus or minus) between the extended fingers and the tip of the toe.
Agility/dynamic balance	8-foot up-and-go test	Number of seconds required to get up from a seated position, walk 8 feet (2.44 meters), turn, and return to seated position on chair.
Aerobic endurance	2-minute step test	Number of full steps completed in 2 minutes, raising each knee to a point midway between the patella (kneecap) and iliac crest (top hip bone).The score is the number of times the right knee reaches the required height).

**Perceived Wellness Survey:** To assess perceived well-being, the Perceived Wellness Survey (PWS) was used. The PWS, designed by Adams, Benzer, Garner, and Woodruff [22], contains a total of 36 items. There are six items for each dimension: mental health (items 1,7,13,19,25,31); emotional health (items 2,8,14,20,26,32); social health (items 3,9,15,21,27,33); physical health (items 4,10,16,22,28,34); spiritual health (items 5,11,17,23,29,35); and intellectual health (items 6,12,18,24,30,36). Each dimension of PWS [23, 24] was represented by six items that are scored from 1 (very strongly disagree) to 6 (very strongly agree). The score for each participant could range from 36 to 216, with higher scores representing a better perception of wellness. The questionnaire of the PWS was first explained to the participants about how to respond to each question and its answering on the Likert scale, and if necessary, explanations were provided. All participants completed the PWS after completing the SFT.

## Statistical Analysis

Statistical analyses were performed using the SAS (Statistical Analysis System) statistical package (version 9.0; SAS Institute, Inc., Cary, NC). The descriptive statistics are presented as means and standard deviations for continuous variables and as frequencies and percentages for categorical variables used for all measures. Inferential statistic was developed using analysis of variance (ANOVA). Bivariate correlation analysis for individual test of physical fitness and composite wellness score and between component of wellness and physical fitness test of elderly participants were also calculated. Multiple linear regressions were used to analyze the relationship of wellness composite score with physical fitness test of elderly study participants. The statistical significance was set at p< 0.05.

## Results

A total of 400 elderly participants comprising 284 men and 116 women with a mean age of 69.80±3.82 years and 67.25±2.57 years, respectively, were assessed for physical fitness tests and perceived wellness. The maximum number of subjects was in the age group of 65−69 years. Based on marital status, 84%were married. Almost half (49%) of participants completed university degree and only 7.25 percent were uneducated. Most of the participants (55.50% males and 21.75% females) stayed in joint families, and 58.25% of participants were economically independent. The baseline characteristics of the participants are shown in Table 2.

**Table 2.** Socio-demographics variables of the elderly participants

**Parameter**	**No. of participants**		
	**Total**	**Males**	**Females**
Age Group(in years), n (%) , mean±SD			
65–69	247 (61.75)	147 (36.75) 67.41 ± 1.29	100 (25) 66.44 ± 1.39
70–74	131 (32.75)	117 (29.25) 71.04 ± 1.28	14 (3.5) 71.50 ± 1.22
75–79	14 (3.5)	12 (3) 76.83 ± 1.27	2 (0.5) 78.00 ± 0
≥80	8 (2)	8 (2) 85.00 ± 5.61	-
Marital status, n (%)			
Single	7 (1.75)	5 (1.25)	2 (0.50)
Married	336 (84)	242 (60.50)	94 (23.50)
Widow/Separated/Divorced	57 (14.25)	37 (9.25)	20 (5)
Education, n (%)			
Uneducated	29 (7.25)	10 (2.50)	19 (4.75)
Primary school	51 (12.75)	29 (7.25)	22 (5.50)
Secondary school	124 (31)	86 (21.50)	38 (9.50)
University	196 (49)	159 (39.75)	37 (9.25)
Type of family, n (%)			
Nuclear	91 (22.75)	62 (15.50)	29 (7.25)
Joint	309 (77.25)	222 (55.50)	87 (21.75)
Economic status, n (%)			
Dependent	167 (41.75)	74 (18.50)	93 (23.25)
Independent	233 (58.25)	210 (52.50)	23 (5.75)

n(%) = number (percentage); SD = standard deviation.

Among the elderly males, strength testing for ‘arm curl’ test showed lower mean value in the 75–79 year age-group compared to the other age groups. ‘Chair stand’ test showed a linear decrease in strength in the age groups with the 75–79 years age-group having a lowest value of 8.83±1.26. Flexibility testing showed that ‘chair sit and reach’ test values were higher than ‘back scratch’ test in the age-groups of 70–74, 75–79 and≥80 years, indicating lower limb flexibility was better than upper limb among these age groups, except in the 65–69 year age-group. The ‘8-feet up and go’ test showed linear increase in mean values with advancing age, indicating age related decline in agility and dynamic balance with age. The‘2-minute step’ test showed a linear decrease in mean scoreswith advancing age. With consideration of statistical significance among the elderly men participants, body mass index, chair stand test, back scratch test, ‘8-foot up & go’ test and ‘2-minute step’ test showed a statistical significance with *p* < 0.05,while ‘arm curl’ test and ‘chair sit & reach’ test showed no significance [Table 3].

**Table 3.** Test scores of the physical fitness tests of elderly male and female participants

**TEST (Unit)**	**Gender**	**Age group and number of elderly participants**				**P-value**
		**65–69 years** **Men, n=147** **Women, n=100**	**70–74 years** **Men, n=117** **Women, n =14**	**75–79 years** **Men, n=12** **Women, n=8**	**80 years & above** **Men, n=8** **Women, n =0**	
**BMI (Kg/m^2^)**	Men Women	23.4±2.11 21.3±3.39	22.9 ± 1.76 23.3 ± 3.50	22.8 ± 3.18 22.9 ± 00	21.8 ± 1.30 -	0.039^*^ 0.101
**Arm curl test (no.)**	Men Women	11.4 ± 3.71 14.5 ± 4.63	11.4 ± 3.89 12.2 ± 2.43	9.58 ± 4.03 7.00 ± 0	10.7 ± 2.60 -	0.407 0.017^*^
**Chair stand test (no.)**	Men Women	15.2 ± 4.64 13.6 ± 4.26	13.5 ± 4.30 9.9 ± 2.01	8.83 ± 1.26 5.00 ± 0	9.37 ± 1.18 -	<.0001^*^ 0.0002^*^
**Back scratch test (cm)**	Men Women	-7.41 ±7.96 -9.8 ± 7.28	10.5 ± 9.11 -13.2 ± 8.88	-8.75 ± 7.68 -34.0 ± 0	-20.2 ±14.6 -	<.0001^*^ <.0001^*^
**Chair sit and reach test (cm)**	Men Women	-13.7 ± 7.22 -8.4 ± 6.92	13.4 ± 8.91 16.5 ± 11.2	9.83 ± 7.09 -24.0 ± 0	18.6 ± 5.37 -	0.112 <.0001^*^
**8-foot up & go test (seconds)**	Men Women	7.26 ± 1.46 8.0 ± 2.03	7.68 ± 1.69 11.6 ± 5.20	9.22 ± 2.87 20.3 ± 0	13.9 ±- 4.11 -	<.0001^*^ <.0001^*^
**2-minute step test (no.)**	Men Women	67.4 ± 21.9 62.7 ± 16.9	56.1 ± 21.4 51.7 ± 10.5	53.4 ± 12.2 37 ± 0	47.2 ± 14.0 -	<.0001^*^ 0.0085^*^

n = number of participants, SD= standard deviation, kg/m2 = kilogram/meter square, no.= number, cm=centimeter, Statistical significance, p < 0.05

Test scores of the physical fitness in elderly females are shown in Table 3. The ‘arm curl’ test showed highest values in the 65–69 years age group, while the lowest values were seen in the 75–79 years age group. In ‘chair stand’ test, the maximum values were recorded in the 65–69 year age-group, and the minimum values were recorded in the 75–79 year age-group. In the ‘back scratch & chair sit’ andin the ‘reach’ tests better flexibilities were recorded in the 65–69 yearage-group. Maximum time to perform the ‘8-feet up and go’ test was noted in 75–79 year age-group, whereas the minimum time was recorded in the 65–69 year age-group. The 65–69 years age group performed the ‘2-minute step’ test with a mean of 62.7 ± 16.9 while those in the age group ≥80 years performed the least (37 ± 0). The entire senior fitness test except body mass index among the elderly women participants showed a statistical significance with *p* < 0.05.

With results of statistical analysis for age-group wise comparison among elderly men and women, in ‘arm curl’ test, elderly women performed superior in 65–69, 70–74 years age-group in comparison to men participants whereas 75–79 years elderly men scored highest mean values in upper body strength testing. In lower body strength testing with chair stand test, elderly men had highest values in all the 65–69, 70–74 and 75–79 years group. Similarly, for flexibility testing with back scratch test, elderly men had better flexibility with minimum values, whereas in chair sit and reach test 70–74, 75–79 years women shown less flexibility compared to men participants. Maximum time to perform ‘8-foot up and go’ test was noted among the elderly women participants in the age-group of 65–69, 70–74 and 75–79 years respectively, indicating agility/dynamic balance were better among the elderly men participants who perform the test in minimum time. Aerobic endurance testing with ‘2-minute step’ test showing better performance among the elderly men as on comparison to women participants.

Wellness composite score of elderly males (Table 4) were maximum in the75–79 year age-group with a mean of 14.54±2.31, and minimum score of 11.91±1.66 was recorded in the 65–69 year group. Wellness components like emotional wellness showed minimum mean scoresof 2.04±0.42 in 65-69 years age-group, and psychological wellness showed a maximum mean score of 3.13±0.41 in the 75–79 year age-group. Intellectual wellness and physical wellness components showed progressive increase in mean scores with advancing age. In elderly men, component of perceived wellness like intellectual, psychological, social and spiritual wellness’s showed no statistical significance, while emotional, physical and composite wellness score wellness had statistical significance with *p* < 0.05. Among elderly females (Table 4), the 70–74 year age-group recorded a maximum mean score of 15.26±2.29 in the wellness composite score. Wellness components like social wellness showed maximum mean score of 3.34±0.30 in 70–74 years age group, whereas minimum mean values were recorded in spiritual wellness with a mean of 0.71±0 in 75–79 year group. Social wellness (3.34 ± 0.30), psychological wellness (3.30±0.32), spiritual wellness (3.16±0.42) and intellectual wellness (3.12±0.61) in 70–74 year showed maximum mean values for component of perceived wellness on comparisons to other age-groups. Among the elderly women participants, composite wellness score and component of perceived wellness like physical, social and spiritual wellness showed a statistical significance with *p* < 0.05, whereas emotional, intellectual, psychological wellness component had no significance statistically.

**Table 4.** Perceived wellness survey scores of elderly male and female participants

**Component of wellness**	**Gender**	**Age-group and number of elderly participants**				**P-value**
		**65–69 years** **Men, n=147** **Women, n=100**	**70–74 years** **Men, n=117** **Women, n =14**	**75–79 years** **Men, n=12** **Women, n=8**	**80 years & above** **Men, n=8** **Women, n =0**	
**Emotional wellness**	Men Women	2.04 ± 0.42 2.50 ± 0.51	2.19 ± 0.54 2.75 ± 0.55	2.86 ± 0.42 2.29 ± 0	2.59 ± 0.46 -	<.0001^*^ 0.190
**Intellectual wellness**	Men Women	2.85 ± 0.35 2.97 ± 0.42	2.85 ± 0.41 3.12 ± 0.61	2.88 ± 0.45 2.78 ± 0	3.10 ± 0.18 -	0.704 0.827
**Physical wellness**	Men Women	2.33 ± 0.49 2.63 ± 0.46	2.40 ± 0.55 2.93 ± 0.48	2.69 ± 0.58 3.07 ± 0	2.97 ± 0.58 -	0.003^*^ <.0001^*^
**Psychological wellness**	Men Women	2.88 ± 0.21 3.01 ± 0.37	2.88 ± 0.29 3.30 ± 0.32	3.13 ± 0.41 3.00 ± 0	2.90 ± 0.43 -	0.148 0.280
**Social Wellness**	Men Women	2.98 ± 0.26 2.93 ± 0.46	2.94 ± 0.33 3.34 ± 0.30	3.03 ± 0.58 2.91 ± 0	2.90 ± 0.36 -	0.582 0.001^*^
**Spiritual wellness**	Men Women	2.84 ± 0.34 3.00 ± 0.49	2.84 ± 0.36 3.16 ± 0.42	3.11 ± 0.51 0.71 ± 0	2.78 ± 0.50 -	0.075 <.0001^*^
**Wellness Composite**	Men Women	11.91±1.66 13.45 ± 1.86	12.10 ± 1.80 15.26 ± 2.29	14.54±2.31 9.14 ± 0	13.55±0.86 -	0.015^*^ 0.035^*^

n = number of participants, SD= standard deviation ,Statistical significance, p < 0.05

Correlation analysis of physical fitness test with perception of wellness (composite score) of elderly participants (Table 5) showed a statistical significant association (p< 0.05) of ‘arm curl’ test (p=0.012), ‘back scratch’ test (p=0.0002), ‘8-feet up and go’ test (p=0.005), ‘2-minute step’ test (p=0.005) with the composite wellness score among the elderly males, whereas among elderly females only ‘2 minute step’ test (p= 0.007) was highly significant with the wellness score. In all participants, both male and female, except for BMI (p=0.805) and ‘chair stand’ test (p=0.627) which showed no significant association with wellness score, all other fitness tests were strongly associated with the composite wellness score.

**Table 5.** Bivariate correlation between physical fitness test and wellness composite score of the elderly participants

**Pearson correlation coefficients** **Prob>IrI**	**Wellness composite score**		
**Test item** **(Senior fitness test)**	**Elderly males** **n=284**	**Elderly females** **n=116**	**Total(male and female)** **n=400**
Body mass index	-0.03942	0.12151	0.01235
	0.5082	0.1938	0.8055
Arm curl test	0.147	0.120	0.119
	0.012^*^	0.199	0.016^*^
Chair stand test	-0.080	0.101	-0.024
	0.173	0.297	0.627
Back scratch test	-0.221	-0.011	- 0.158
	0.0002^*^	0.905	0.001^*^
Chair sit and reach test	-0.062	-0.163	- 0.099
	0.291	0.080	0.046^*^
8-foot up and go test	0.163	0.069	0.114
	0.005^*^	0.457	0.021^*^
2-minute step test	0.165	0.246	0.184
	0.005^*^	0.007^*^	0.0002^*^

n = number of participants

With bivariate correlation of physical fitness test and component of perceived wellness among the entire elderly participants (Table 6), a statistically significant association was found between component emotional wellness with the entire physical fitness test except for BMI (p=0.835) and ‘2-minute step’ test (p=0.068). The p-values for the component physical and psychological wellness showed a significant association with ‘arm curl’ test, ‘chair stand’ test, ‘back scratch’ test and ‘8- feet up and go’ tests. Intellectual wellness was significantly associated with ‘back scratch’ and ‘8-feet up and go’ tests. Spiritual wellness component showed no statistical correlation with BMI, ‘chair stand’, ‘back scratch’, ‘chair sit and reach’ and ‘2-minute step’ tests, whereas social wellness had a significant association with ‘chair stand’, ‘chair sit and reach’ and ‘2-minute step’ tests. Moreover with multiple linear regression analysis, wellness of different subscale and component as independent variables showed that wellness values were significantly associated with the entire physical fitness test with p<0.0001, indicating a strong correlation (Table 7).

**Table 6.** Bivariate correlation between physical fitness test and component of wellness of the all elderly participants

**Component of Wellness**	**Test item of Senior fitness test**						
	**Body mass Index**	**Arm curl test**	**Chair stand test**	**Back scratch test**	**Chair sit and reach test**	**8-Foot up & go test**	**2-minute step test**
Emotional wellness	-0.0104	0.2390	-0.2108	-0.2598	0.1324	0.2323	0.0912
	0.835	<.0001^*^	<.0001^*^	<.0001^*^	0.008^*^	<.0001^*^	0.068
Intellectual wellness	-0.0547	0.0565	-0.0784	-0.1867	-0.0810	0.1265	-0.0176
	0.275	0.258	0.117	0.0002^*^	0.105	0.011^*^	0.724
Physical wellness	0.0399	0.1826	-0.1998	-0.3412	-0.0011	0.3417	0.0598
	0.425	0.0002^*^	<.0001^*^	<.0001^*^	0.981	<.0001^*^	0.232
Psychological wellness	-0.0166	0.1130	-0.1012	-0.0998	0.0179	0.1384	0.0398
	0.739	0.023^*^	0.042^*^	0.045^*^	0.720	0.005^*^	0.427
Social wellness	-0.0078	0.0679	0.1039	0.0178	-0.1268	-0.0029	0.0992
	0.874	0.175	0.037^*^	0.721	0.011^*^	0.953	0.047^*^
Spiritual wellness	-0.0596	0.1475	0.0051	0.0687	0.0789	-0.0966	0.0913
	0.234	0.003^*^	0.917	0.170	0.114	0.053^*^	0.067

**Table 7.** Multiple linear regression between wellness and physical fitness test of all elderly participants

**Parameter Estimates**							
**Variable**	**Parameter Estimate**	**Standard Error**	**t-Value**	**Pr > |t|**	**Standardized Estimate**	**R-Square**	**Adj R-Sq**
Arm curl test	0.93844	0.01601	58.6	<.0001	0.94652	0.8959	0.8956
Chair stand test	0.81249	0.01633	49.75	<.0001	0.92799	0.8612	0.8608
Back scratch test	- 0.74508	0.03213	- 23.19	<.0001	- 0.7577	0.5741	0.573
Chair sit and reach test	- 0.69858	0.02445	- 28.58	<.0001	- 0.81961	0.6718	0.6709
8-Foot up and go test	1.44958	0.02269	63.88	<.0001	0.95443	0.9109	0.9107
2-Minute step test	0.18495	0.0033	56.05	<.0001	0.94197	0.8873	0.887

## Discussion

Functional fitness represents the physical capacity that is needed to undertake normal everyday activities independently and without the early onset of fatigue [25]. However the ageing process tends to reduce physical fitness (strength, endurance, agility and flexibility) and results in difficulties in daily life activities and normal functioning of the elderly [26,27]. Elderly population tends to be less active with advancing age, even though it is well known that regular physical activity is important for independent living, prevention of chronic health problems and quality of life [28-30]. Evidence suggests that being physically active can help elderly person to strengthen bones and muscles, improve their ability to do daily activities, prevent falls and reduce the risks of chronic disease, depression, control weight and increase life expectancy [31]. The present study analysed the physical fitness and perceived wellness of functionally independent community dwelling elderly for four age subgroups: 65–69 years, 70–74 years, 75–79 years and ≥80 years. The most obvious change usually associated with ageing process is that of body composition, with a reduction in total and lean body mass. While there are no standard cut-offs of BMI in elderly, BMI of 20–25 are generally considered to be in the healthy range, with those above or below this range can be associated with diseases or functional limitations [32,33]. Wei L, Wu B (2014), in a longitudinal analysis study examined the racial/ethnic differences in the effects of BMI on the onset of functional impairment over 10 years of follow-up and concluded that risk of developing ADL difficulty was higher for Hispanics than for Blacks in the obese category and for the onset of mobility difficulty, no significant differences were found across racial/ethnic groups within any BMI category [34]. However, BMI of our study participants was in the optimal range indicating their adequate nutritional status.

Usually elderly people tend to be less flexible and our results of flexibility testing showed that elderly women were less flexible than elderly men of the same age group, thereby reflecting some gender differences accompanying ageing, probably resulting fromsarcopenia which also involves the loss of muscle functionality leading to mobility restriction, functional impairment and physical disability [35]. Moreover, in menopausal women there is a physiological decline, particularly a reduction in bone mineral density (BMD), muscle mass, strength that can be attributed to estrogen deficiency [36]. In elderly men (aged 70–74 years), muscle strength testing with ‘arm curl’ test and ‘chair stand’ test showed maximum mean of 11.4 ± 3.89 and 15.2 ± 4.64 (65–69 years).In women, the 65–69 years group showed maximum values of 14.5 ± 4.63 in ‘arm curl’ test and 13.6 ± 4.26 in ‘chair stand’ test. Decrease in muscle strength during the ageing process is result of significant loss and atrophy of muscle cells, which may hamper the physical activity level and increase the risk of falls in elderly. In an cross-sectional study Alcazar J et al. (2021) found annual percentage losses in relative muscle power to increase progressively from the age of 30 years (~1%) to the oldest age (~6–9%) in a European cohort population. This implies muscle power as one of the primary therapeutic targets for resistance training interventions in elderly population to prevent or revert physical disability [37].

Assessment of agility and dynamic balance testing (the ‘8-feet up and go’ test) showed better performance score. A decreased time to perform the test signifies improvement, for example, faster speed to complete the ‘8-feet up and go’ test and that was observed only in 65–69 years age-group, whereas the scores increased as age progressed. A linear decline in agility and dynamic balance and aerobic endurance testing performance score were noted among the elderly population. This may be due to decreased rate of maximal oxygen consumption (VO_2_ max), which is not constant throughout life, but has been shown to accelerate significantly with each decade increase in age [38].

Since physical fitness is an outcome of physical activity participation, therefore the increased prevalence of cardiovascular and musculoskeletal diseases may be an impending factor for the practice of physical activities on the part of the elderly [39] and that could be the probable reason behind the low level of fitness among the elderly population. In an observatory study by Anjana et al. (2014) on physical activity and inactivity patterns in India where it was observed that physical inactivity was significantly more common among the people of urban section of the country and most of the inactive subjects were from the elderly population [40]. Nevertheless, it is important to systematically assess the functional/physical fitness of elderly people, which may allow for the preparation of intervention, treatment, care and rehabilitation programs [41].

Healthy ageing refers to a multi-dimensional concept involving a variety of physical, psychological, socio-cultural and spiritual factors [42]. While analyzing which component of wellness improve as age advances it was found that intellectual and physical wellness ascends in elderly men participants, whereas among elderly women only the physical wellness seems to be better as one ages. The most probable reason regarding with improved physical wellness among the elderly women may be that most of the age related changes in physical health occur in the early sixth and seventh decade of life. Physical activity choices, physiological, psycho-social and environmental determinants of activity participation vary by the socio-demographic characteristics of age, sex and education level [43,44] and this could be the reason behind variation in wellness score. Emotional wellness showing minimum scores as compared to other components of wellness among elderly participants indicating emotional changes may be a pre-dominant factor in wellness variation in elderly population of this region of the country.

While interpreting results of correlation test for perceived wellness score with physical fitness test, it was found that fitness test such as BMI, upper and lower body strength and flexibility, agility and dynamic balance test were not statistically significant with composite wellness score in elderly women, except for aerobic endurance (p = 0.007) which had a strong association. This finding was in contrast to elderly men, where upper body strength and flexibility, agility and dynamic balance and aerobic endurance testing showed significant correlation with the wellness score. However, for entire elderly population, the correlation values showed that BMI and lower body strength were not significantly associated with the wellness scores. The most obvious outcome of the current study showed that BMI and lower body strength test appears to be the poor predictor for well-being, whereas aerobic endurance test (‘2-minute step’ test) being the best predictor of wellness for the elderly in the study. While in a survey study by Lijing L Yan et al. (2004), it was reported that both underweight and obese older adult had impaired quality of life, particularly worse physical functioning and physical well-being [45].

After analyzingthe association between the individual component of wellness and individual test of physical fitness it was found that emotional wellness had the strongest association with the entire physical fitness test except for BMI and aerobic endurance test. This finding suggests that the emotional wellness component is an important determinant or health protective factor in maintaining overall physical fitness. On further analysis, it was found that values of the components of wellness and physical fitness tests were strongly correlated and dependent on each other. Additionally, the findings of our study suggest that high wellness is a strong predictor for improved physical fitness among the elderly which may create an opportunity for primary geriatric care to develop subject-specific intervention for their successful ageing.

Therefore, assessment of physical related fitness and well-being stands as an important measure in order to promote healthy ageing. A prescription of appropriately tailored basic exercises should be included in all healthcare recommendations in an effort to enhancefunctional independence, psychological well-being, and quality of life through the promotion of exercise for all elderly individuals of any age, whether fit or frail [46,47].

There were certain limitations of the present study. First, the community dwelling elderly participants were selected from a limited geographical area. Second, only urban elderly population was considered for the study. Third, screening for cognitive impairment and chronic health conditions (morbidity pattern), assessment for level of physical activity and their associations with physical fitness and perceived well-being of elderly were not considered in this study. Fourth, from a purely quantitative and statistical approach, laboratory based physical fitness testing for the elderly could have generated more valid results and outcome which may help/assist to predict the decline in functional capacity among them.

## Conclusions

Screening of physical fitness and wellness were found to be important measures in assessing wellness of community dwelling elderly population, and in predicting their overall state of well-being, including age-specific comparison of fitness performance and wellness score.This community based study was confined to a select geographical region of the country. A larger epidemiological study in an extended geographical region may be helpful to generalize these results for the overall benefit of the elderly population of this region.
